# Performance of Ceftazidime-Avibactam 30/20-μg and 10/4-μg Disks for Susceptibility Testing of *Enterobacterales* and Pseudomonas aeruginosa

**DOI:** 10.1128/spectrum.02720-22

**Published:** 2023-02-06

**Authors:** Renru Han, Siquan Shen, Dandan Yin, Li Ding, Qingyu Shi, Yang Yang, Yan Guo, Shi Wu, Peiyuan Zhi, Demei Zhu, Fupin Hu

**Affiliations:** a Institute of Antibiotics, Huashan Hospital, Fudan University, Shanghai, China; b Key Laboratory of Clinical Pharmacology of Antibiotics, Ministry of Health, Shanghai, China; Institut National de Santé Publique du Québec

**Keywords:** ceftazidime-avibactam, disk diffusion test, *Enterobacterales*, *Pseudomonas aeruginosa*

## Abstract

Ceftazidime-avibactam, a new β-lactam–β-lactamase inhibitor combination, is active against multidrug-resistant *Enterobacterales* and Pseudomonas aeruginosa isolates and has became available for clinical use in China in the latter half of 2019. In this study, we evaluated the performance of the disk diffusion test with ceftazidime-avibactam 10/4-μg and 30/20-μg disks, compared with the reference broth microdilution method, with a collection of 467 *Enterobacterales* and 182 P. aeruginosa nonduplicate clinical isolates. The results of antimicrobial susceptibility testing indicated that the categorical agreement (CA) of ceftazidime-avibactam 10/4-μg disk testing for all tested *Enterobacterales* isolates was 99.8%, with 0.5% very major errors (VMEs) and no major error (ME). The CA of ceftazidime-avibactam 10/4-μg disk testing for all tested P. aeruginosa isolates was 87.9%, with 15.5% MEs and no VME. The CA of ceftazidime-avibactam 30/20-μg disk testing for all tested *Enterobacterales* isolates was 99.4%, with 1.5% VMEs and no ME. The CA of ceftazidime-avibactam 30/20-μg disk testing for all tested P. aeruginosa isolates was 91.8%, with 2.5% VMEs and 9.9% MEs. Overall, ceftazidime-avibactam 10/4-μg disk testing showed superior performance and was more suitable for assessment of the susceptibility of *Enterobacterales* and P. aeruginosa isolates.

**IMPORTANCE** Multidrug-resistant *Enterobacterales* and P. aeruginosa strains have become a global public threat, with the emergence and prevalence of plasmid-mediated extended-spectrum β-lactamases (ESBLs), AmpC cephalosporinases, and carbapenemases disseminated worldwide. Ceftazidime-avibactam, which is commercially available, has shown excellent *in vitro* activity against multidrug-resistant and carbapenem-resistant *Enterobacterales* and P. aeruginosa isolates. Moreover, ceftazidime-avibactam has shown promise in treating infections caused by multidrug-resistant and carbapenem-resistant isolates. The disk diffusion test for ceftazidime-avibactam is the most common antimicrobial susceptibility testing method in most laboratories in China. The accurate detection of ceftazidime-avibactam susceptibility is of great significance for the rational clinical application of drugs. Here, we evaluated the performance of the ceftazidime-avibactam 10/4-μg and 30/20-μg disk diffusion tests, compared with the reference broth microdilution method, with clinical *Enterobacterales* and P. aeruginosa isolates.

## INTRODUCTION

Multidrug-resistant *Enterobacterales* and Pseudomonas aeruginosa isolates have increased rapidly in past decades, with the emergence and prevalence of plasmid-mediated extended-spectrum β-lactamases (ESBLs), AmpC cephalosporinases, and carbapenemases disseminated worldwide, which have become a global public threat (1, 2). The emerging resistance to first-line antimicrobial agents, such as β-lactams and fluoroquinolones, has limited the options for empirical treatment for clinicians (1, 3, 4). Ceftazidime-avibactam has shown excellent *in vitro* activity against ESBL-producing isolates, AmpC-producing isolates, class A β-lactamase-producing isolates, and some class D β-lactamase-producing isolates, excluding class B β-lactamase-producing isolates (1, 5, 6). Moreover, ceftazidime-avibactam has shown promise in treating infections caused by multidrug-resistant and carbapenem-resistant isolates (7–10). Thus, accurate detection of ceftazidime-avibactam susceptibility is of great significance for the rational clinical application of drugs. Concerningly, the ceftazidime-avibactam automated susceptibility system is not widely applied in China, and the reference broth microdilution (BMD) method is difficult for routine microbiology laboratories to perform. Although Etest has demonstrated exemplary performance for ceftazidime-avibactam susceptibility testing with *Enterobacterales* and P. aeruginosa isolates (11–13), it is costly. To sum up, the disk diffusion test is the optimal option in most laboratories, because it is convenient, economical, and practical; however, there are some drawbacks to the disk diffusion test, such as different specifications and manufacturers, subjective measurements, and inoculum effects. Ceftazidime-avibactam 10/4-μg and 30/20-μg disks are recommended for antimicrobial susceptibility testing (AST) by the European Committee on Antimicrobial Susceptibility Testing (EUCAST) and the Clinical and Laboratory Standards Institute (CLSI), respectively. Here, we evaluated the performance of the two ceftazidime-avibactam disk diffusion tests, compared with the reference BMD method, for clinical *Enterobacterales* and P. aeruginosa isolates.

## RESULTS

**FIG 1:**
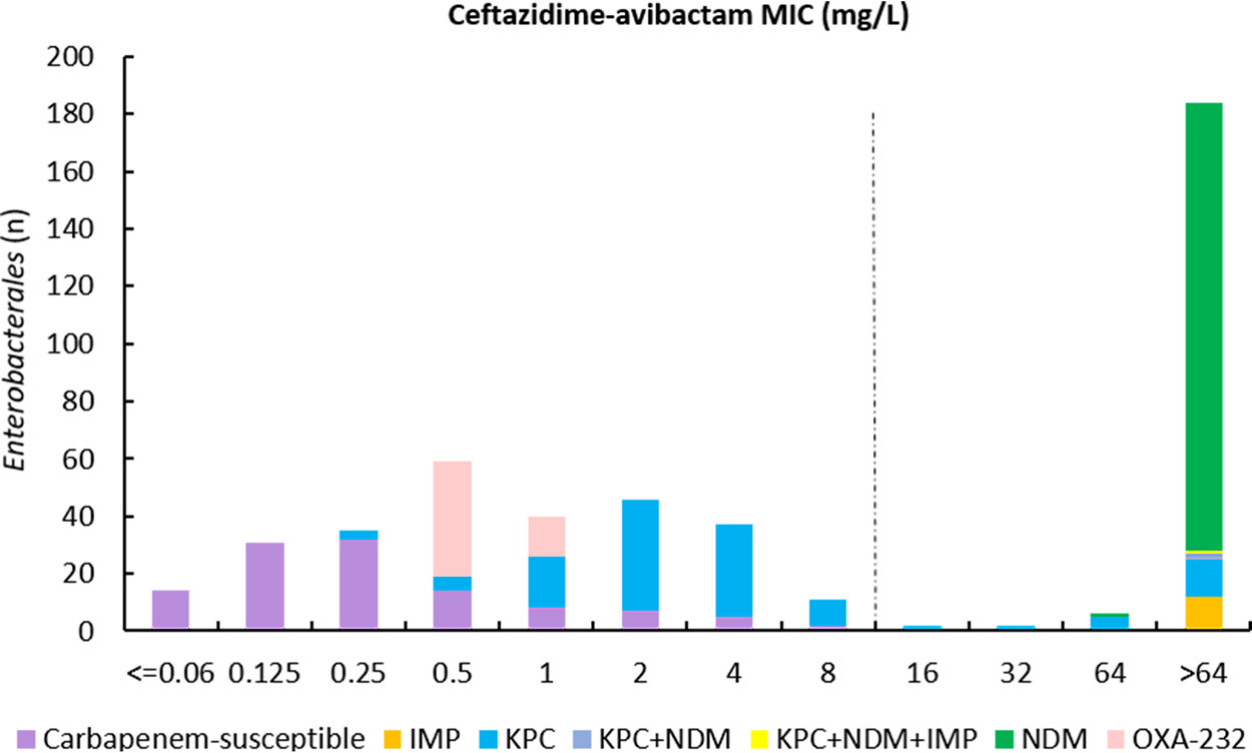
Ceftazidime-avibactam MIC distribution for 467 *Enterobacterales* isolates. The dashed line indicates EUCAST and CLSI breakpoints for ceftazidime-avibactam.

**FIG 2:**
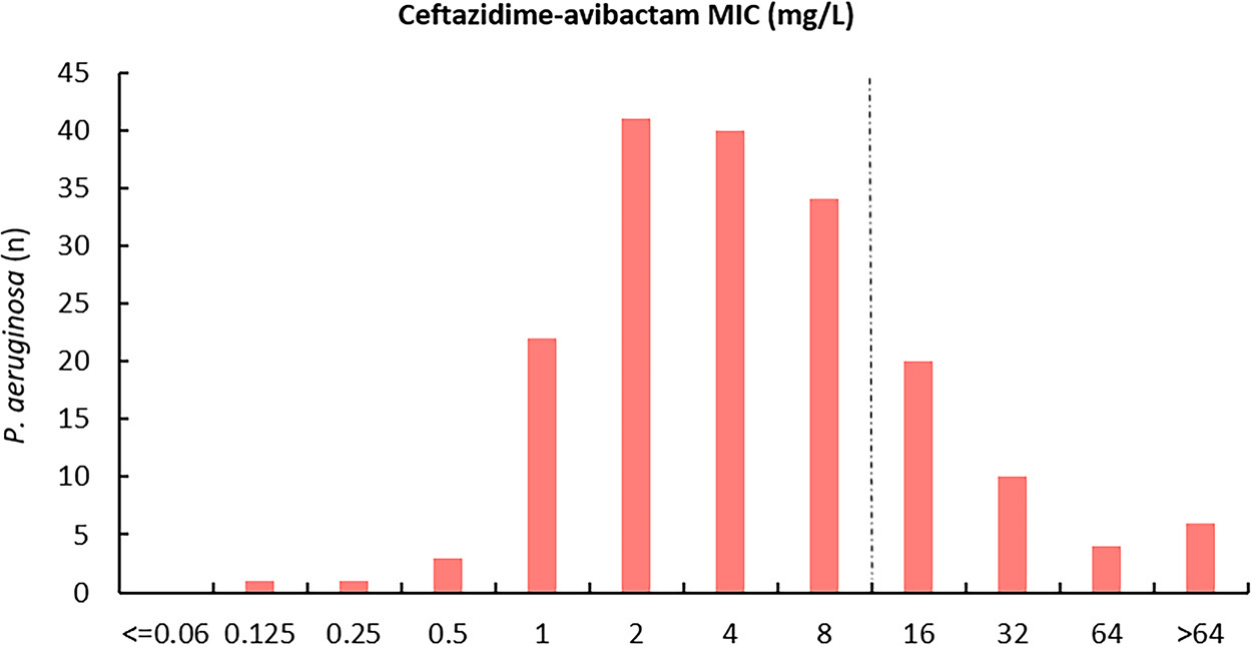
Ceftazidime-avibactam MIC distribution for 182 P. aeruginosa isolates. The dashed line indicates EUCAST and CLSI breakpoints for ceftazidime-avibactam.

In this study, about 75.2% of *Enterobacterales* isolates (351/467 isolates) were carbapenemase-producing isolates and 24.8% (116/467 isolates) were carbapenemase-negative isolates (Table 1). The results of the BMD method indicated that 41.5% of *Enterobacterales* isolates (194/467 isolates) (Fig. 1) and 22.0% of P. aeruginosa isolates (40/182 isolates) (Fig. 2) were resistant to ceftazidime-avibactam.

**TABLE 1:** Detection of carbapenemase genes among 467 *Enterobacterales* isolates

Species	Carbapenemase gene (no. of isolates)
Klebsiella pneumoniae (*n* = 210)	*bla*_KPC-2_ (90), *bla*_KPC-33_ (4), *bla*_KPC-35_ (2), *bla*_KPC-71_ (1), *bla*_KPC-76_ (8), *bla*_KPC-78_ (1), *bla*_KPC-79_ (1), *bla*_OXA-232_ (54), *bla*_NDM-1_ (7), *bla*_NDM-5_ (2), *bla*_IMP_ (3), *bla*_KPC+NDM_ (1), carbapenemase-negative (36)
Escherichia coli (*n* = 176)	*bla*_KPC-2_ (10), *bla*_NDM-1_ (20), *bla*_NDM-5_ (84), *bla*_NDM-4_ (1), *bla*_NDM-9_ (1), *bla*_NDM-13_ (1), *bla*_IMP_ (1), carbapenemase-negative (58)
Enterobacter cloacae (*n* = 54)	*bla*_KPC-2_ (6), *bla*_NDM-1_ (20), *bla*_NDM-5_ (9), *bla*_IMP_ (7), carbapenemase-negative (12)
Klebsiella aerogenes (*n* = 10)	*bla*_KPC-2_ (2), *bla*_NDM-1_ (2), *bla*_NDM-5_ (1), carbapenemase-negative (5)
Klebsiella oxytoca (*n* = 7)	*bla*_KPC-2_ (3), *bla*_NDM-5_ (1), *bla*_KPC+NDM_ (1), *bla*_KPC+NDM+IMP_ (1), carbapenemase-negative (1)
Citrobacter freundii (*n* = 5)	*bla*_KPC-2_ (1), *bla*_NDM-1_ (2), *bla*_NDM-5_ (2)
Providencia rettgeri (*n* = 3)	*bla*_KPC-2_ (1), *bla*_NDM-1_ (1), carbapenemase-negative (1)
Proteus mirabilis (*n* = 1)	Carbapenemase-negative (1)
Serratia marcescens (*n* = 1)	Carbapenemase-negative (1)

### Correlation between ceftazidime-avibactam 10/4-μg disk diffusion test and BMD results.

**FIG 3:**
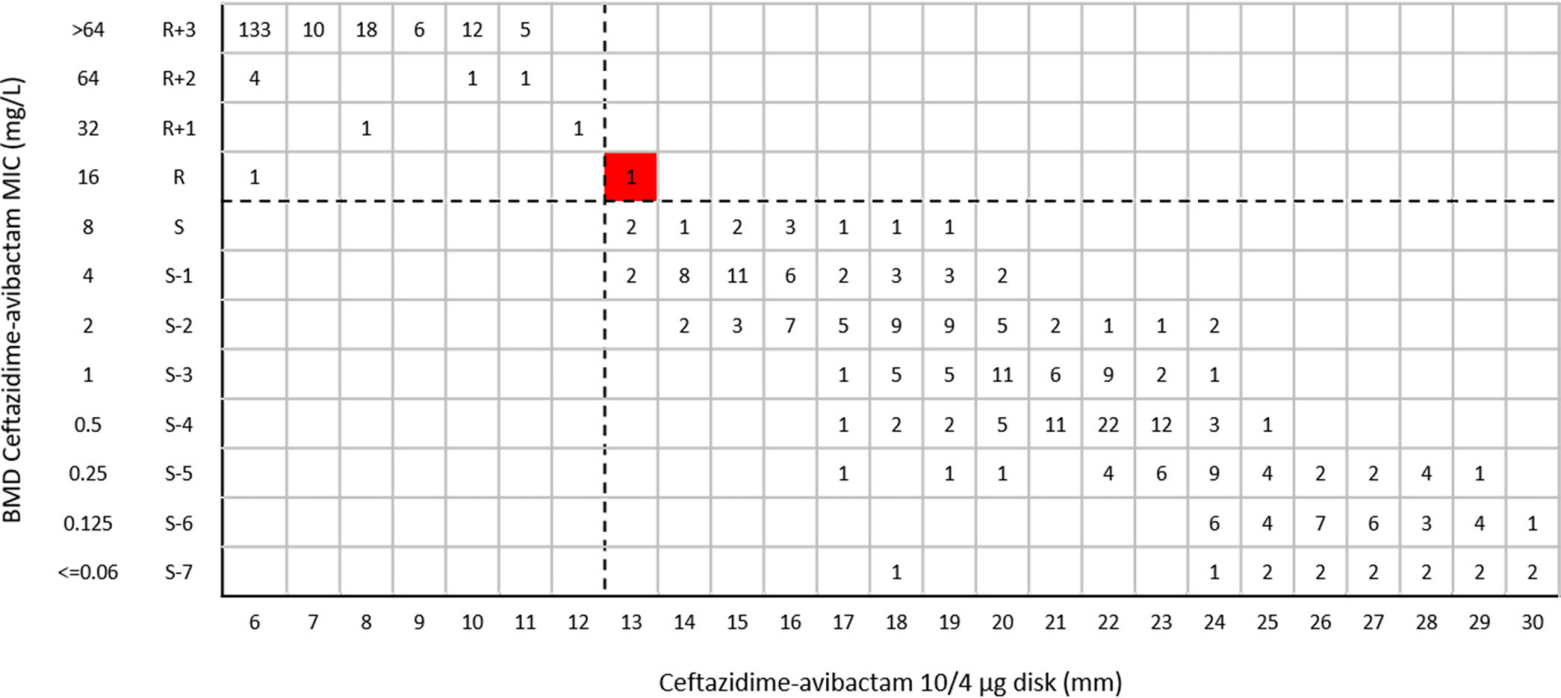
Scattergram comparing ceftazidime-avibactam BMD MIC values and disk diffusion zone diameters with 10/4-μg disks among *Enterobacterales* isolates (*n* = 467). Dashed lines indicate ceftazidime-avibactam breakpoints (EUCAST). The red background indicates that a VME occurred for the disk diffusion method, compared with BMD.

**FIG 4:**
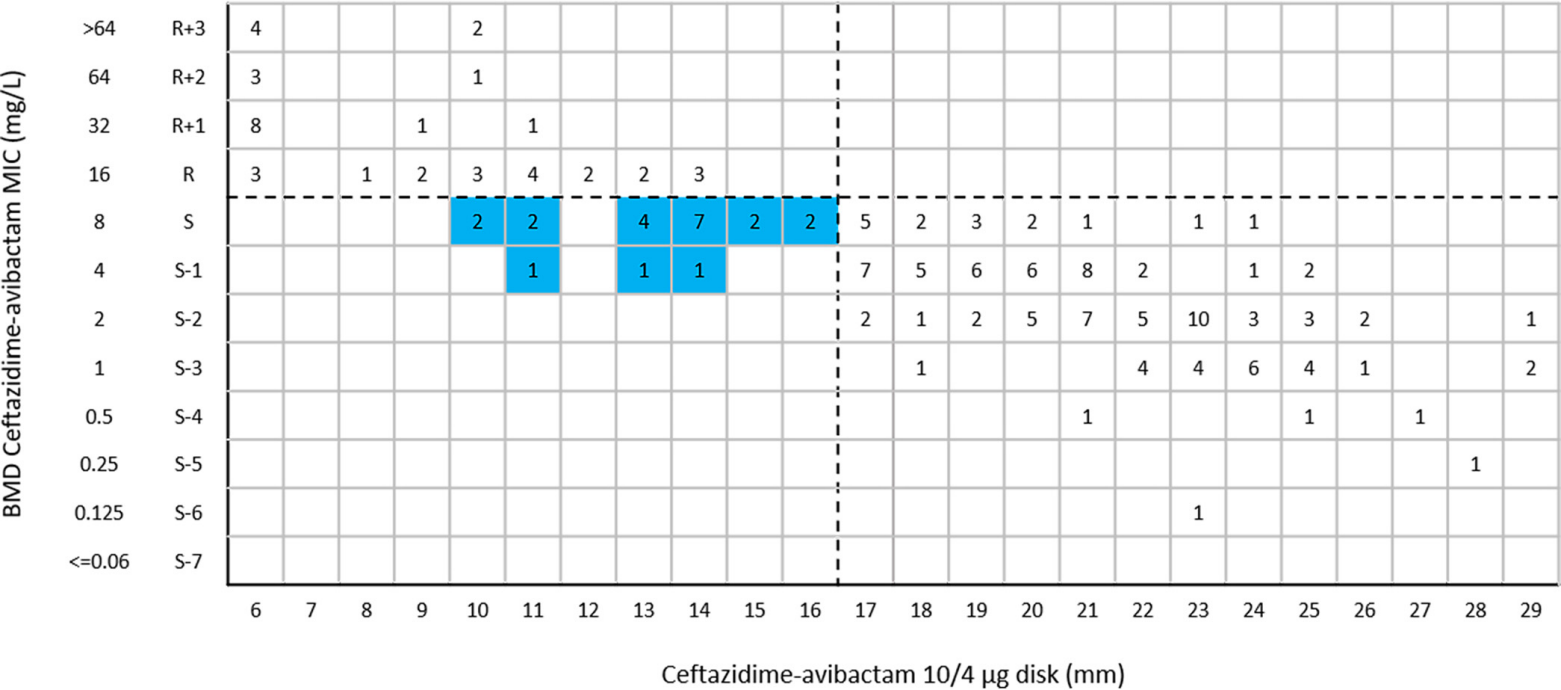
Scattergram comparing ceftazidime-avibactam BMD MIC values and disk diffusion zone diameters with 10/4-μg disks among P. aeruginosa isolates (*n* = 182). Dashed lines indicate ceftazidime-avibactam breakpoints (EUCAST). The blue background indicates that MEs occurred for the disk diffusion method, compared with BMD.

The categorical agreement (CA) of ceftazidime-avibactam 10/4-μg disk test results for all tested *Enterobacterales* isolates was 99.8%, with 0.5% very major errors (VMEs) (Table 2). The CA, VME rate, and major error (ME) rate for the ceftazidime-avibactam 10/4-μg disk test results for *bla*_KPC_-positive *Enterobacterales* clinical isolates (*n* = 126) were 99.2%, 5.0%, and 0%, respectively. The CA values for the ceftazidime-avibactam 10/4-μg disk test results for *bla*_MBL_-positive (*n* = 168), *bla*_OXA-232_-positive (*n* = 54), and carbapenemase-negative *Enterobacterales* clinical isolates (*n* = 116) were all 100%, and no VME or ME were observed (Table 2). One K. pneumoniae isolate harboring *bla*_KPC-2_ was considered a VME; it was resistant to ceftazidime-avibactam with a MIC of 16 mg/L but was susceptible by the ceftazidime-avibactam 10/4-μg disk diffusion test, with an inhibitory zone of 13 mm (Fig. 3). The CA of ceftazidime-avibactam 10/4-μg disk test results for all tested P. aeruginosa isolates was 87.9%, with 15.5% MEs and no VME (Table 2). Twenty-two P. aeruginosa isolates were susceptible to ceftazidime-avibactam with MICs of 4 or 8 mg/L by BMD but were resistant by the ceftazidime-avibactam 10/4-μg disk diffusion test, with inhibitory zones of 10 to 16 mm (Fig. 4).

**TABLE 2:** Performance of ceftazidime-avibactam 10/4-μg and 30/20-μg disk diffusion tests versus reference BMD in testing the susceptibility of *Enterobacterales* and P. aeruginosa isolates

Species and isolate type	Ceftazidime-avibactam 10/4-μg disk test results			Ceftazidime-avibactam 30/20-μg disk test results		
	CA (% [no. of isolates])	VME (% [no. of isolates])	ME (% [no. of isolates])	CA (% [no. of isolates])	VME (% [no. of isolates])	ME (% [no. of isolates])
All *Enterobacterales* isolates (*n* = 467)	99.8 (466)	0.5 (1)	0 (0)	99.4 (464)	1.5 (3)	0 (0)
*bla*_KPC_-positive isolates (*n* = 126)	99.2 (125)	5.0 (1)	0 (0)	99.2 (125)	5.0 (1)	0 (0)
*bla*_MBL_-positive isolates (*n* = 168)	100 (168)	0 (0)	0 (0)	98.8 (166)	1.2 (2)	0 (0)
*bla*_OXA-232_-positive isolates (*n* = 54)	100 (54)	0 (0)	0 (0)	100 (54)	0 (0)	0 (0)
Carbapenemase-negative isolates (*n* = 116)	100 (116)	0 (0)	0 (0)	100 (116)	0 (0)	0 (0)
P. aeruginosa isolates (*n* = 182)	87.9 (160)	0	15.5 (22)	91.8 (167)	2.5 (1)	9.9 (14)

### Correlation between ceftazidime-avibactam 30/20-μg disk diffusion test and BMD results.

**FIG 5:**
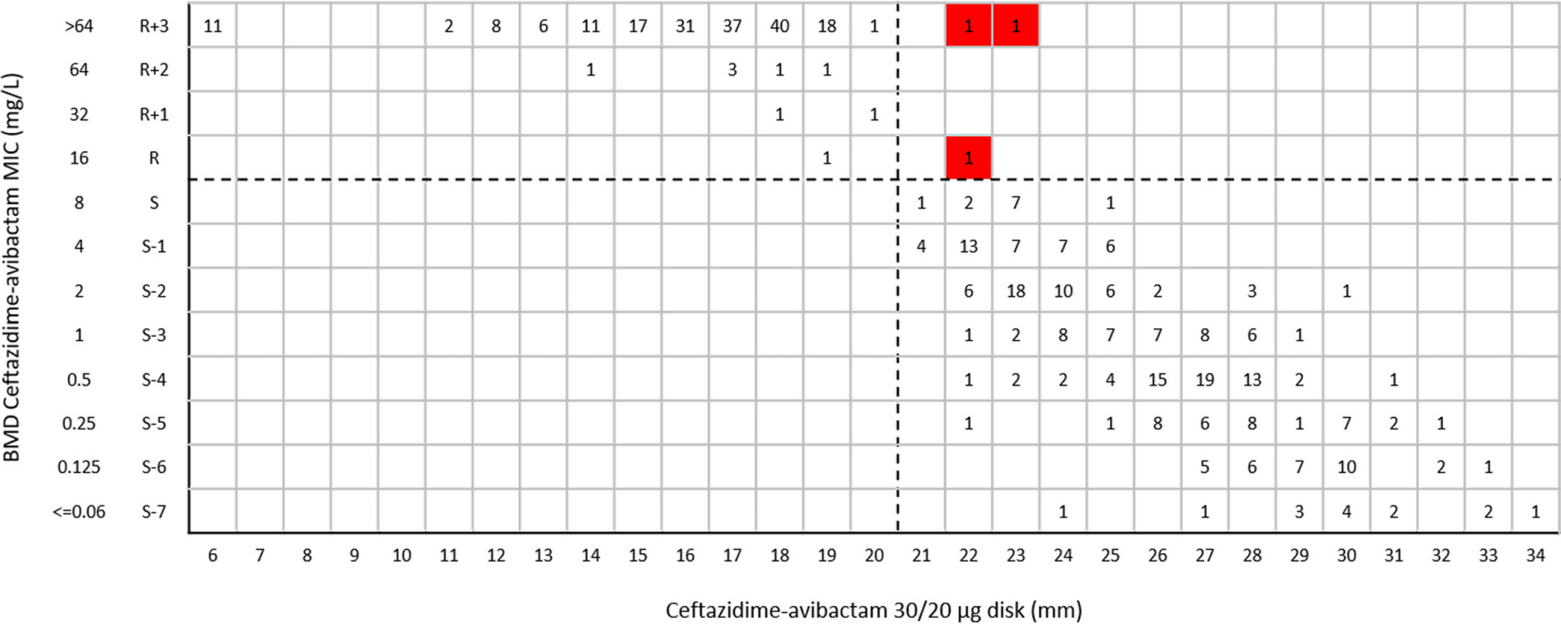
Scattergram comparing ceftazidime-avibactam BMD MIC values and disk diffusion zone diameters with 30/20-μg disks among *Enterobacterales* isolates (*n* = 467). Dashed lines indicate ceftazidime-avibactam breakpoints (CLSI). The red background indicates that VMEs occurred for the disk diffusion method, compared with BMD.

**FIG 6:**
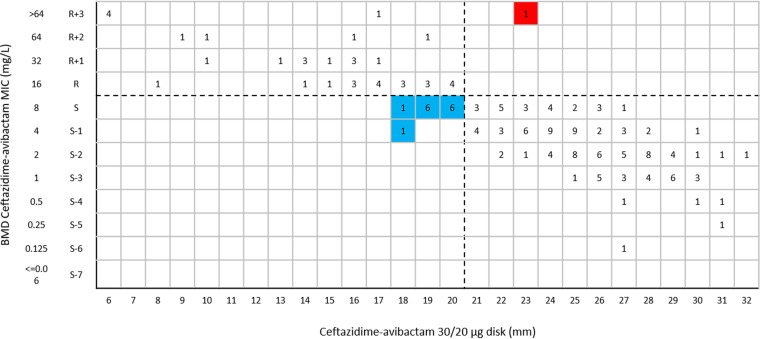
Scattergram comparing ceftazidime-avibactam BMD MIC values and disk diffusion zone diameters with 30/20-μg disks among P. aeruginosa isolates (*n* = 182). Dashed lines indicate ceftazidime-avibactam breakpoints (EUCAST). The red background indicates that a VME occurred for the disk diffusion method, compared with BMD. The blue background indicates that MEs occurred for the disk diffusion method, compared with BMD.

The CA of ceftazidime-avibactam 30/20-μg disk test results for all tested *Enterobacterales* isolates was 99.4%, with 1.5% VMEs (Table 2). The CA values for ceftazidime-avibactam 30/20-μg disk test results for *bla*_KPC_-positive and *bla*_MBL_-positive isolates were 99.2% and 98.8%, respectively, and the VME rates were 5.0% and 1.2%, respectively (Table 2). The CA values for ceftazidime-avibactam 30/20-μg disk test results for *bla*_OXA-232_-positive and carbapenemase-negative *Enterobacterales* clinical isolates were both 100%, with no VME or ME (Table 2). One K. pneumoniae isolate harboring *bla*_KPC-2_ was resistant to ceftazidime-avibactam with a MIC of 16 mg/L but was susceptible by the ceftazidime-avibactam 30/20-μg disk diffusion test, with an inhibitory zone of 22 mm (Fig. 5). Two Escherichia coli isolates harboring *bla*_NDM_ were resistant to ceftazidime-avibactam with a MIC of >64 mg/L by BMD but were susceptible by the ceftazidime-avibactam 30/20-μg disk diffusion test, with inhibitory zones of 22 mm and 23 mm (Fig. 5). The CA of ceftazidime-avibactam 30/20-μg disk test results for all tested P. aeruginosa isolates was 91.8%, with 2.5% VMEs and 9.9% MEs (Table 2). One P. aeruginosa isolate was resistant to ceftazidime-avibactam with a MIC of >64 mg/L but was susceptible by the ceftazidime-avibactam 30/20-μg disk diffusion test, with an inhibitory zone of 23 mm (Fig. 6). Fourteen P. aeruginosa isolates were susceptible to ceftazidime-avibactam by BMD, with MICs of 4 or 8 mg/L, but were resistant by the ceftazidime-avibactam 30/20-μg disk diffusion test, with inhibitory zones of 18 to 20 mm (Fig. 6).

In this study, the ceftazidime-avibactam 10/4-μg disk inhibitory zones of 16 P. aeruginosa isolates (8.8% [16/182 isolates]) were 16 to 17 mm, situated in the area of technical uncertainty (ATU), which needed to be addressed before reporting; the confirmatory MIC results showed that all of these isolates had MICs of 2 to 8 mg/L (Fig. 4). When tested with ceftazidime-avibactam 30/20-μg disks, 7.1% of *Enterobacterales* isolates (33/467 isolates) had inhibitory zones of 20 to 22 mm; the confirmatory MIC results showed that 2 of the 33 isolates represented false-susceptible results, with MICs of >64 mg/L and 16 mg/L but both with inhibitory zones of 22 mm (Fig. 5). In brief, the parameters of the ceftazidime-avibactam 10/4-μg and 30/20-μg disk tests with the tested *Enterobacterales* isolates were in line with the criteria for acceptability (VME rates should be <1.5%, and ME rates should be <3%). The ME rate (15.5%) for the ceftazidime-avibactam 10/4-μg disk test with the tested P. aeruginosa isolates was unacceptable according to the CLSI criteria (ME rates should be <3%). The ME (9.9%) and VME (2.5%) rates for the ceftazidime-avibactam 30/20-μg disk test with the tested P. aeruginosa isolates were both below the mark according to the CLSI criteria.

## DISCUSSION

**FIG 7:**
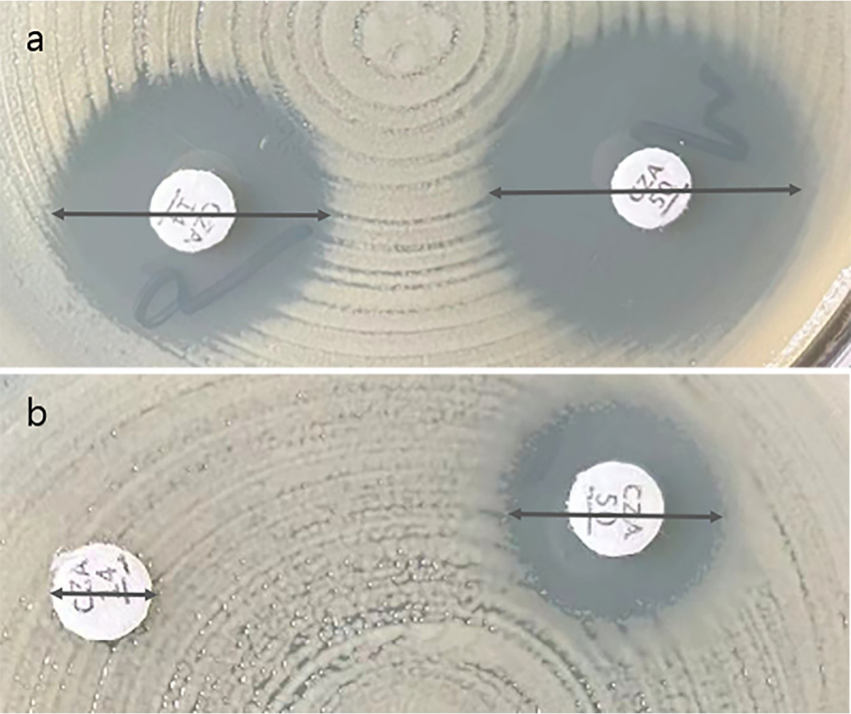
Ceftazidime-avibactam disk diffusion test results for K. pneumoniae isolates. (a) 10/4-μg disk inhibitory zone (22 mm) (left) and 30/20-μg disk inhibitory zone (28 mm) (right), with MIC of 1 mg/L. (b) 10/4-μg disk inhibitory zone (6 mm) (left) and 30/20-μg disk inhibitory zone (14 mm) (right), with MIC of >64 mg/L.

**FIG 8:**
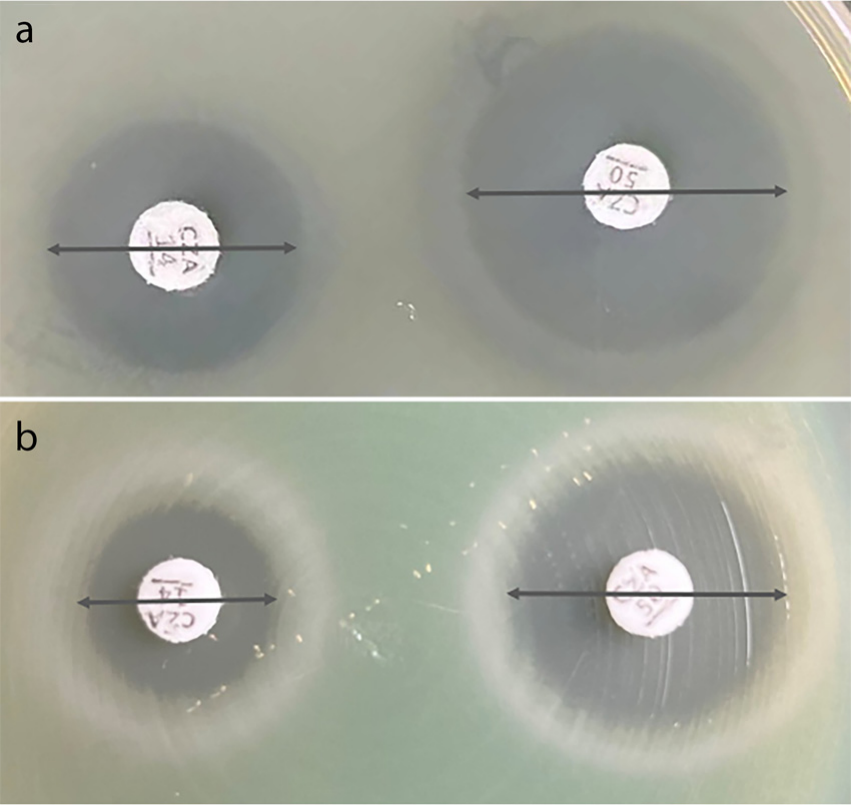
Ceftazidime-avibactam disk diffusion test results for P. aeruginosa isolates. (a) 10/4-μg disk inhibitory zone (17 mm) (left) and 30/20-μg disk inhibitory zone (23 mm) (right), with MIC of 8 mg/L. (b) 10/4-μg disk inhibitory zone (14 mm) (left) and 30/20-μg disk inhibitory zone (20 mm) (right), with MIC of 8 mg/L.

**FIG 9:**
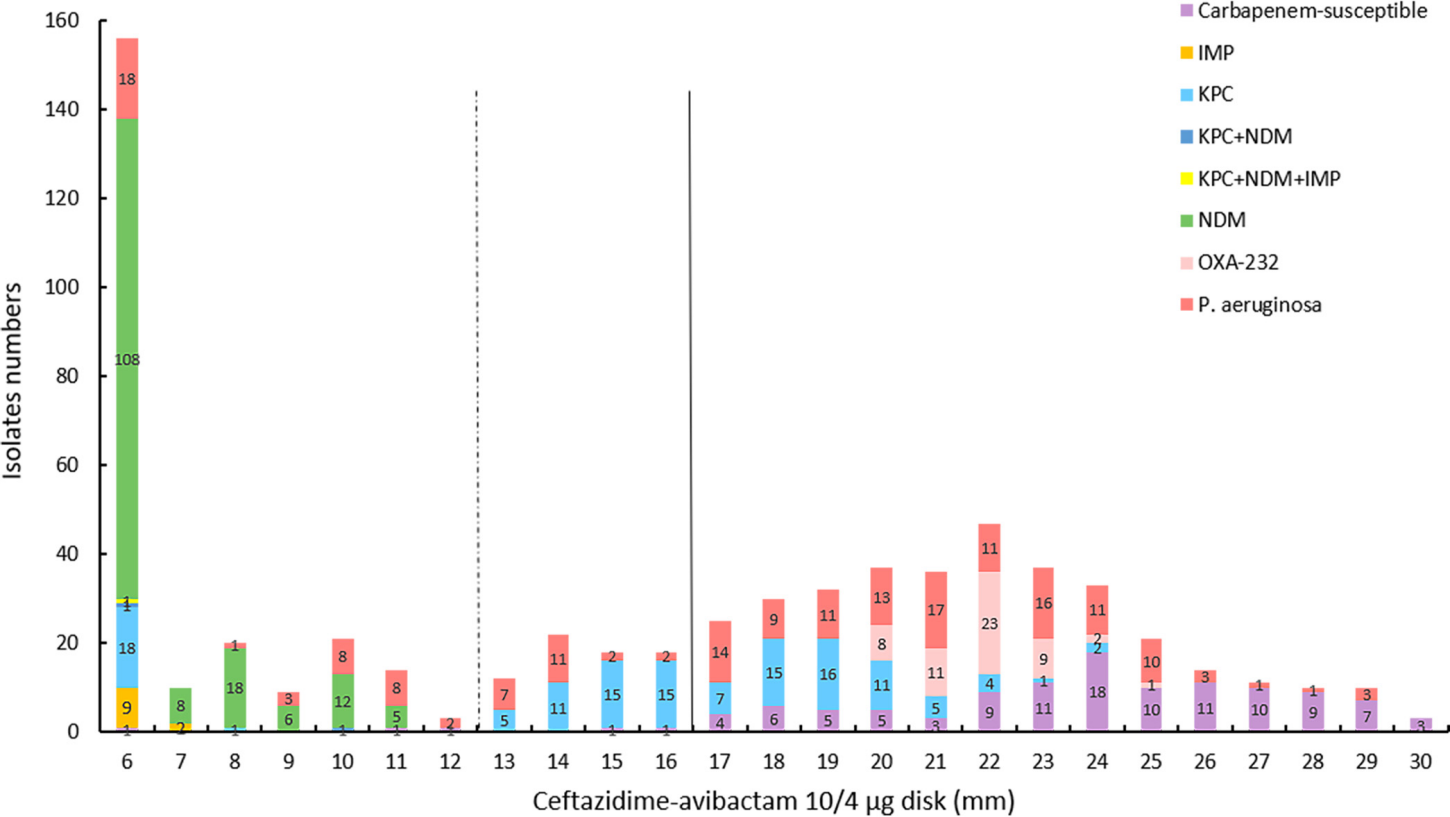
Ceftazidime-avibactam 10/4-μg disk inhibitory zone distribution for tested *Enterobacterales* (*n* = 467) and P. aeruginosa (*n* = 182) isolates. The dotted line and solid line indicate the ceftazidime-avibactam breakpoints for *Enterobacterales* and P. aeruginosa clinical isolates, respectively, according to the EUCAST.

**FIG 10:**
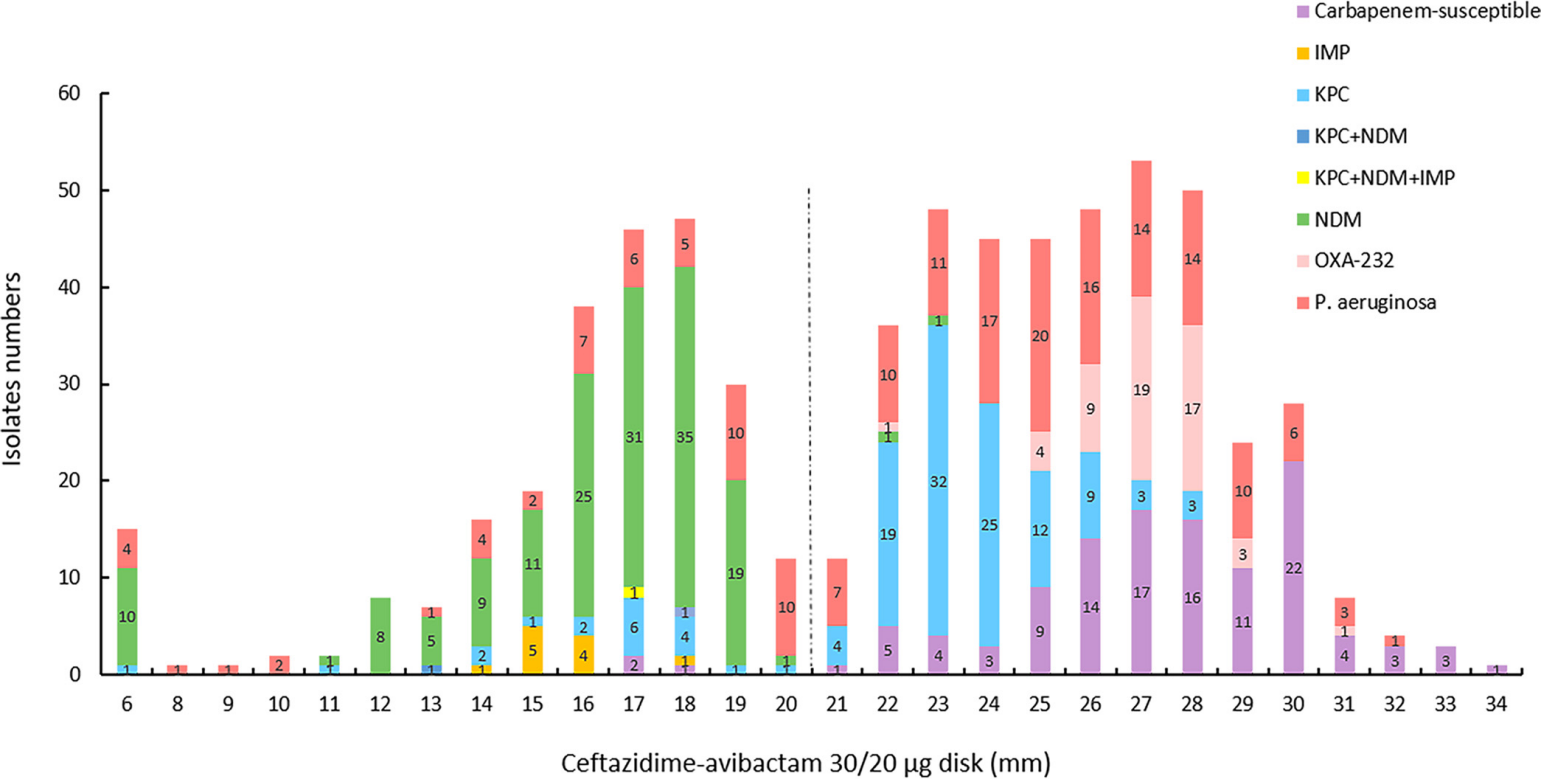
Ceftazidime-avibactam 30/20-μg disk inhibitory zone distribution for tested *Enterobacterales* (*n* = 467) and P. aeruginosa (*n* = 182) isolates. The dotted line indicates the ceftazidime-avibactam breakpoint for *Enterobacterales* and P. aeruginosa clinical isolates according to the CLSI.

The two ceftazidime-avibactam disk diffusion tests with *Enterobacterales* isolates reached 95% CA, and the VME rates (0.5% and 1.5%) were acceptable (13, 14). The ceftazidime-avibactam 10/4-μg disk diffusion test performed well with the tested *Enterobacterales* isolates (CA, 99.8%) and P. aeruginosa isolates (CA, 87.9%), but a high ME rate (15.5%) occurred with P. aeruginosa isolates. The ceftazidime-avibactam 30/20-μg disk diffusion test also showed good performance with the tested *Enterobacterales* isolates (CA, 99.4%) and P. aeruginosa isolates (CA, 91.8%), while high ME (9.9%) and VME (2.5%) rates were noted with the P. aeruginosa isolates. The CA, VME rate, and ME rate for the subclassified carbapenemase-producing isolates make no difference in this study. Similar results were reported for disk diffusion tests in previous studies (CA, 79.7 to 96.4%) and there were few VMEs (0 to 4.8%), but high ME rates (2.9% to 30.9%) occurred in all of those studies, warning about an overestimation of ceftazidime-avibactam resistance in P. aeruginosa clinical isolates (15–19). Therefore, the reference BMD method or other alternative methods should be performed to avoid reporting false-resistant results. Compared with the ceftazidime-avibactam 10/4-μg disk, the inhibitory zones of the ceftazidime-avibactam 30/20-μg disk showed more large zones for resistant isolates (≤20 mm) with an unclear distinction (Fig. 7 and 8). As observed in this study, the 30/20-μg disk inhibitory zones of ceftazidime-avibactam-resistant *Enterobacterales* isolates were mainly distributed at 14 to 19 mm (83.5%), while 10/4-μg disk results were mainly distributed at 6 to 8 mm (86.1%) (Fig. 9 and 10). In addition, the measurement results of the inhibitory zones were subjective and visually misinterpreted, which affected the ceftazidime-avibactam susceptibility results.

Notably, KPC variants showing ceftazidime-avibactam resistance have been reported in different regions of the world, commonly with high MICs, which lead to limited therapeutic options and should be fully evaluated (20–23). The two ceftazidime-avibactam disk diffusion tests showed excellent consistency with BMD for *bla*_MBL_-positive isolates (including those with *bla*_NDM_, *bla*_IMP_, *bla*_KPC+NDM_, and *bla*_KPC+NDM+IMP_) and *bla*_KPC-variant_-positive isolates, which were all resistant to ceftazidime-avibactam by BMD. The *bla*_KPC-variant_-, *bla*_IMP_-, and *bla*_NDM_-harboring isolates were resistant to ceftazidime-avibactam, consistent with the previous study in China (6). Ceftazidime-avibactam has been used in clinical practice for a long period, and the breakpoint criteria for the 30/20-μg disk diffusion test still need to be optimized, especially for *Enterobacterales* isolates, because their resistance may be overestimated with inhibition zones between 18 and 20 mm in the 2019 CLSI M100 document (24). In 2020, CLSI stipulated that, for *Enterobacterales* isolates, the confirmatory MIC testing is indicated for isolates with ceftazidime-avibactam inhibition zone diameters of 20 to 22 mm, to avoid reporting false-susceptible or false-resistant results (25). The ME rate for the ceftazidime-avibactam 30/20-μg disk diffusion test was 9.9% for the P. aeruginosa isolates, and the inhibitory zones were distributed at 18 to 20 mm. We suggest that the confirmatory MIC testing should also be indicated for P. aeruginosa clinical isolates with ceftazidime-avibactam inhibition zone diameters of 18 to 20 mm in CLSI documents, to avoid reporting false-susceptible or false-resistant results. In addition, the EUCAST needs to extend the ATU range for P. aeruginosa clinical isolates, because high ME rates usually occurred for inhibition zone diameters of 10 to 15 mm. However, the confirmatory MIC testing for a number of isolates would limit the application of ceftazidime-avibactam 30/20-μg disk testing in some laboratories, especially in low-resource areas. Alternative economical commercial AST methods, like the Vitek 2 automatic dilution system (CA, 99.2% to 100%) and Etest (CA, 99.6% to 100%), for ceftazidime-avibactam should be in clinical use at an early date, but the commercial Vitek 2 automatic dilution method for ceftazidime-avibactam has become available relatively recently and systematic performance assessments have not yet been widely published, especially data from China (18, 26–28).

There are a few limitations in this study. The carbapenemases in *Enterobacterales* isolates were subdivided into four subclasses, because the antimicrobial spectrum of ceftazidime-avibactam covers class A β-lactamases, class C β-lactamases, and some class D β-lactamases but excludes class B β-lactamases. However, the presence of carbapenemases or other resistance mechanisms in P. aeruginosa isolates were not screened in this study. The group of ceftazidime-avibactam-resistant P. aeruginosa clinical isolates was not large enough. Another limitation of this study is that these disks from only one manufacturer were tested; because of the ceftazidime-avibactam 10/4-μg disk scarcity, only disks from the manufacturer the MAST Group in the United Kingdom seem to be available, and disks from other companies are unavailable. To use uniform material to ensure comparability of results, we used both ceftazidime-avibactam 10/4-μg disks and ceftazidime-avibactam 30/20-μg disks from the specific manufacturer. In the future, disks from more manufacturers should be tested (if available) to more comprehensively assess the consistency of results between different manufacturers' disks.

Based on our study, the two ceftazidime-avibactam disk diffusion tests performed well against the tested *Enterobacterales* isolates (CA, 99.8% and 99.4%), while VMEs (2.5%) with ceftazidime-avibactam 30/20-μg disks occurred in P. aeruginosa clinical isolates. Moreover, high ME rates occurred in both ceftazidime-avibactam 10/4-μg disks (15.5%) and ceftazidime-avibactam 30/20-μg disks (9.9%). According to the EUCAST breakpoint, P. aeruginosa isolates with the inhibitory zones of 16 to 17 mm, situated in the ATU, need to be assessed before reporting, to avoid reporting false-susceptible or false-resistant results. Overall, we thought that the ceftazidime-avibactam 10/4-μg disk was more suitable than the ceftazidime-avibactam 30/20-μg disk for assessment of the susceptibility of *Enterobacterales* and P. aeruginosa isolates.

## MATERIALS AND METHODS

### Clinical strains.

A total of 467 nonduplicate *Enterobacterales* isolates, including Klebsiella pneumoniae (*n* = 210), Escherichia coli (*n* = 176), Enterobacter cloacae (*n* = 54), Klebsiella aerogenes (*n* = 10), Klebsiella oxytoca (*n* = 7), Citrobacter freundii (*n* = 5), Providencia rettgeri (*n* = 3), Proteus mirabilis (*n* = 1), Serratia marcescens (*n* = 1), and 182 nonduplicate P. aeruginosa isolates, were collected from the China Antimicrobial Surveillance Network (CHINET) between January 2017 and February 2022. Species were identified using a matrix-assisted laser desorption ionization–time of flight mass spectrometry (MALDI-TOF MS) system (bioMérieux, France) according to the manufacturer's instructions. These clinical *Enterobacterales* isolates were mainly collected from among carbapenem-resistant isolates to obtain a sample size large enough for comprehensive evaluation of the performance of ceftazidime-avibactam disks with different carbapenemase-producing isolates. These clinical isolates were mainly collected from the intensive care unit (31.7%), outpatient and emergency departments (9.1%), geriatric department (7.1%), infectious disease department (6.5%), general surgery department (6.2%), neurosurgery department (4.6%), respiratory medicine department (4.2%), urological surgery department (3.5%), neurology department (1.8%), nephrology department (1.7%), neonatology department (1.5%), hematology department (1.5%), and other departments (20.6%). These clinical isolates were isolated from sputum (45.9%), urine (23.0%), blood (7.1%), bronchoalveolar lavage fluid (4.0%), pus (2.6%), secreta (2.8%), ascites (2.2%), bile (1.8%), cerebrospinal fluid (1.4%), drainage fluid (1.4%), wounds (1.2%), and other sources (7.0%).

### AST.

The disk diffusion test with ceftazidime-avibactam 30/20-μg disks (MAST Group, UK) and 10/4-μg disks (MAST Group) was performed in parallel with the reference BMD method according to CLSI guidelines (29). Diameters of inhibition zones (in millimeters) were all measured by two operators. Isolates with ceftazidime-avibactam MICs of ≤8/4 μg/mL were considered susceptible, while those with MICs of ≥16/4 μg/mL were deemed resistant, according to the CLSI and EUCAST breakpoints (30, 31). Isolates with inhibitory zone diameters of ≥21 mm with ceftazidime-avibactam 30/20-μg disks were considered susceptible, while those with values of ≤20 mm were considered resistant by CLSI breakpoints (30). Confirmatory MIC testing needs to be performed for isolates with zone diameters of 20 to 22 mm to avoid reporting false-susceptible or false-resistant results, according to CLSI guidelines. The inhibitory zone diameters with ceftazidime-avibactam 10/4-μg disks for *Enterobacterales* isolates (susceptible, ≥13 mm; resistant, <13 mm) and P. aeruginosa isolates (susceptible, ≥17 mm; resistant, <17 mm) were interpreted with EUCAST breakpoints (31). The E. coli ATCC 25922 and K. pneumoniae ATCC 27853 strains were used for quality control in each test of this study.

### Molecular analysis.

These clinical isolates were screened for the presence of the five most common carbapenemase genes (*bla*_KPC_, *bla*_NDM_, *bla*_IMP_, *bla*_VIM_, and *bla*_OXA-48-like_) by PCR with specific primers and DNA sequencing, as described previously (32).

### Statistical analysis.

The CA indicated the proportion of results in the same interpretive category (susceptible or resistant) with the disk diffusion method and the reference method, using the same reference criteria (14). VME was defined as the proportion of isolates interpreted as susceptible by the disk diffusion test but resistant according to the reference method (14). ME was defined as the proportion of isolates determined as resistant by the disk diffusion test but susceptible by the reference method (14). Rates of less than 1.5% for VME and 3% for ME were acceptable according to CLSI guidelines (14).
